# Reliability, validity, and change over time in disinhibited and disruptive behaviors assessed with the age‐adapted disruptive behavior diagnostic observation schedule in children aged 5–12 years with ADHD

**DOI:** 10.1002/jcv2.70149

**Published:** 2026-09-16

**Authors:** Margreet Bierens, Annick Huberts‐Bosch, Verena Ly, Walter Matthys, Jan Buitelaar, Catharina A. Hartman, Nanda Rommelse

**Affiliations:** ^1^ Karakter Child and Adolescent Psychiatry University Centre Nijmegen the Netherlands; ^2^ Institute of Psychology Leiden University Leiden the Netherlands; ^3^ Department of Clinical Child and Family Studies Utrecht University Utrecht the Netherlands; ^4^ Department of Medical Neuroscience Radboud University Medical Centre Donders Institute for Brain, Cognition and Behavior Nijmegen the Netherlands; ^5^ Department of Psychiatry Interdisciplinary Centre Psychopathology and Emotion Regulation (ICPE) University Medical Centre Groningen University of Groningen Groningen the Netherlands; ^6^ Department of Developmental Psychology Utrecht University Utrecht the Netherlands

**Keywords:** ADHD, change over time, disruptive behavior diagnostic observation schedule, ODD, reliability, validity

## Abstract

**Background:**

Previous research has shown that the disruptive behavior diagnostic observation schedule (DB‐DOS) is a valid and reliable clinic‐based assessment of disinhibited and disruptive behaviors in preschoolers. We adapted the DB‐DOS for use in children aged 5–12 years, examined its psychometric properties, and evaluated its ability to capture change over time across repeated assessments.

**Methods:**

Children (*N* = 212; baseline age *M* = 8.5 years, SD = 2.0; 75.8% boys) with a clinical diagnosis of ADHD participated between 2015 and 2021. Follow‐up data were available for 97% (*N* = 206) at 5 weeks and 75% (*N* = 160) at 1 year. Blinded videotaped sessions were coded by five trained psychologists who had no knowledge of the child, treatment condition, or timing of the observation. Psychometric properties were examined using principal component analysis (PCA), inter‐item correlations, correlations with validated parent and teacher reports, internal consistency estimates, and intraclass correlation coefficients. DB‐DOS change scores at 5 weeks and 1 year were examined in relation to change scores based on parent and teacher reports.

**Results:**

PCA indicated that the correlational structure was captured by two components: Behavioral Regulation (BR; e.g., “Talks excessively,” “Difficulty waiting”) and Anger Modulation (AM; e.g., “Noncompliance,” “Verbal aggression”). Inter‐item correlations and associations between DB‐DOS scores and parent and teacher reports provided partial support for validity, particularly in the parent context (PC). Internal consistency ranged from acceptable to good, and interrater reliability was excellent. Psychometric properties at 5 weeks and 1 year were broadly comparable to those at baseline. Changes in disinhibited and disruptive behaviors observed with the DB‐DOS, particularly in the PC, showed modest associations with changes reported by parents and teachers.

**Conclusion:**

The age‐adapted DB‐DOS showed acceptable to good reliability and provided partial support for validity as a clinic‐based observational tool for assessing disinhibited and disruptive behaviors in children aged 5–12 years with ADHD. Preliminary evidence suggests that the DB‐DOS may capture change over time, particularly in the PC, although change cannot be attributed uniquely to intervention effects. This standardized clinic‐based assessment method holds promise for supporting diagnostic assessment and intervention research in school‐aged children with disinhibited and disruptive behaviors.

## Key Points

**What's known?**

- Structured diagnostic observation of disruptive behavior is rarely used in ADHD assessment, despite being strongly recommended in clinical guidelines.
- Prior work with the disruptive behavior diagnostic observation schedule (DB‐DOS) showed promise in distinguishing normative from clinical disruptive behavior in toddlers.

**What's new?**

- This study adapted the DB‐DOS for 5–12‐year‐olds, demonstrating good reliability and validity, and sensitivity to change over time.
- The age‐adapted DB‐DOS elicited and coded disinhibited and disruptive behaviors, yielding two robust dimensions (Behavioral Regulation and Anger Modulation).

**What's relevant?**

- These findings suggest that the DB‐DOS holds promise for advancing diagnostic practices and can provide a blinded outcome measure in research.

## Graphical abstract

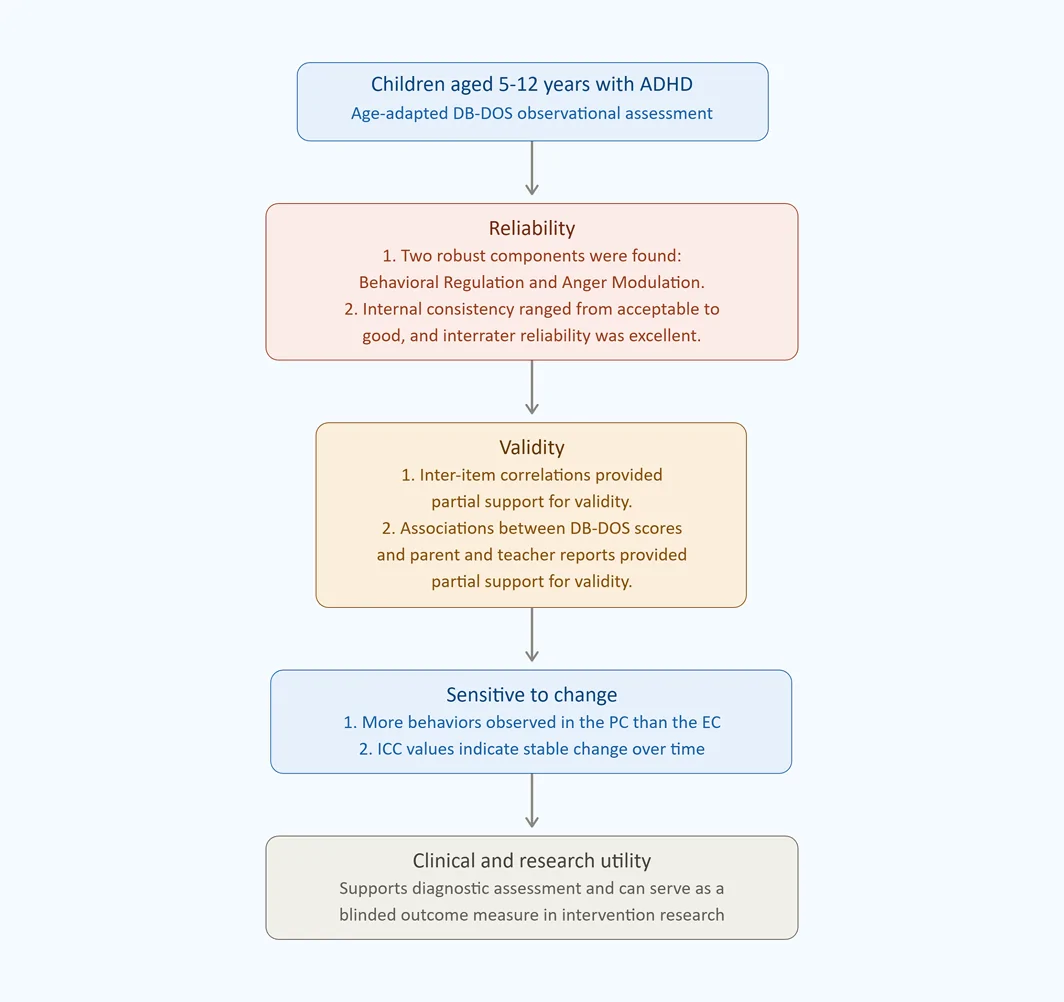

## INTRODUCTION

Within the broader field of neurodevelopmental assessment, ADHD is unusual in that no standardized performance test or structured clinic‐based observation is recommended as part of routine diagnostic decision‐making. Several school observation methods have shown good reliability and validity for ADHD‐related behaviors (e.g., Gadow et al., 1996; Kelman et al., 2024; Shapiro, 2004), but school observations inherently reflect the specific classroom, peer, and teacher context in which they are conducted. A clinic‐based observation can complement such information by standardizing the interactional context and behavioral presses, thereby supporting comparison across children.

The disruptive behavior diagnostic observation schedule (DB‐DOS) was originally developed to elicit and code disruptive behaviors in preschool children, both in interaction with a parent and with an examiner (Wakschlag et al., 2005, Wakschlag, Briggs‐Gowan, et al., 2008, Wakschlag, Hill et al., 2008). Bunte et al. (2013) adapted this approach for Dutch preschool children and extended it with ADHD‐related coding items. Across preschool studies, the DB‐DOS has been shown to capture clinically meaningful variation in BR and AM, with contextual differences between parent‐ and examiner‐based observations (Bunte et al., 2013; Petitclerc et al., 2015). Importantly, the DB‐DOS should not be understood as a stand‐alone diagnostic instrument for ADHD, ODD, or CD. Rather, it provides a standardized observation of disinhibited and disruptive behaviors that are clinically relevant across diagnostic categories.

The DB‐DOS may also be useful when administered repeatedly to examine change over time. Prior studies have used repeated DB‐DOS assessments to map developmental patterns of BR and AM or to evaluate behavioral change in intervention contexts (Diebold et al., 2021; Wiggins et al., 2018; Yarger et al., 2022). Such repeated assessments are particularly relevant when treatment allocation cannot be blinded and parent or teacher ratings may be influenced by informant perspective or expectancy effects. At the same time, changes across repeated assessments should be interpreted cautiously, because they may reflect treatment‐related change as well as natural development, regression to the mean, repeated‐assessment effects, or contextual variation.

To date, there is no established clinic‐based observational tool for assessing disinhibited and disruptive behaviors in school‐aged children. The present study therefore adapted the DB‐DOS for children aged 5–12 years and examined its psychometric properties in a clinical sample of children with ADHD. Specifically, we examined the item structure, internal consistency, interrater reliability, and associations with parent‐ and teacher‐rated behavioral domains. We also evaluated whether the DB‐DOS retained comparable psychometric properties across repeated administrations and whether DB‐DOS change scores corresponded with parent‐ and teacher‐reported change over time.

## METHOD

### Participants

Between 2015 and 2021, a total of *N* = 212 children with ADHD (mean age = 8.5, 76% boys, mean IQ = 100.5 (12.9)) participating in a randomized clinical trial examining nutritional interventions were administered the DB‐DOS before the start of treatment, after 5 weeks and 1 year. Implemented treatments were aimed at reducing disinhibited and disruptive behaviors (Bosch et al., 2020). In addition to a clinical diagnosis established by psychiatrist–psychologist pairs prior to the study, a research diagnosis was made by behavioral scientists and psychologists using a semi‐structured parent interview (Kiddie Schedule for Affective Disorders and Schizophrenia, K‐SADS; Reichart et al., 2011) and a teacher questionnaire (Strengths and Weaknesses of ADHD Symptoms and Normal Behavior Rating Scale, SWAN; Swanson et al., 2012) which included measures of impairment. The inclusion criteria were a confirmation of clinical DSM‐5 ADHD diagnosis (all presentations) with parent and teacher reports (K‐SADS and SWAN). Comorbidities were allowed except for those with eating disorders. A proxy/research diagnosis of ODD was based on the K‐SADS (total score ≥8, with ≥3 items scored as “severe problems”) combined with the conduct problem subscale of the teacher‐reported Strengths and Difficulties Questionnaire (SDQ) (≥4, range 0–10). Probable Autism Spectrum Disorder (ASD) was based on the Children's Social Behavior Questionnaire (CSBQ) and the prosocial behavior subscale of the SDQ, both filled out by parents and teachers. Specifically, probable ASD was defined as a score equal to or higher than the cut‐off score for both raters on at least one of the measures. Probable internalizing disorder was defined as a score equal to or higher than the cut‐off score (≥5, range 0–10) on the emotional symptoms' subscale of the SDQ for at least one of both raters; 37% met criteria for ODD, 17% for ASD, and 34% for internalizing disorders based on scores above a clinical cut‐off (Bosch et al., 2020). Exclusion criteria were non‐confirmation of a clinical DSM‐5 ADHD diagnosis, inability to adhere to dietary treatment (e.g., medical conditions), and severely unstable family situations. The descriptive characteristics of the participants are shown in Table 1.

**TABLE 1:** Sample characteristics at baseline, 5‐week follow‐up, and 1‐year follow‐up.

Characteristic	Category	Baseline	5 weeks	1 year
Child	*N*	215	206	161
Gender	Boys/girls	163/52	155/51	126/35
Age	M (SD)	8.5 (2.0)	8.5 (1.9)	8.4 (1.8)
IQ	M (SD)	100.5 (12.9)	101.1 (13.1)	101.7 (13.1)
ADHD presentation	Combined	118 (55.6)	116 (56.5)	89 (55.2)
	Inattentive	68 (32.0)	66 (32.1)	52 (32.3)
	Hyperactive/impulsive	12 (5.6)	11 (5.3)	10 (6.2)
	Not otherwise specified	14 (6.6)	12 (5.8)	10 (6.1)
Parent				
Mother country of birth	Netherlands/other	181/15	179/16	137/15
Father country of birth	Netherlands/other	176/20	172/23	134/18

### Procedures

The study was approved by the local medical ethics Committee on Research involving Human Subjects (CMO), approval number: 2014‐1349. This trial was registered in the Dutch trial registry (number NTR5434). This study was approved by the Institutional Review Board of Karakter Child and Adolescent Psychiatry and Radboudumc. Written informed consent was obtained, and the questionnaires were filled out before the meeting. Parents received financial compensation for travel costs, and children received a small gift.

Children were invited to participate at three time points: at baseline, after 5 weeks and after 1 year. Children who did not participate in the follow‐up assessments did not differ significantly from those who did on baseline measures. For a detailed description of this study, see (Bosch et al., 2020). Parents were briefed in advance of the DB‐DOS by a trained DB‐DOS performer (*N* = 7). The examination lasted ∼60 min and was performed in a quiet room with a one‐way mirror and a video camera placed behind the screen. Videos were stripped of any information that indicated the type of treatment or time point before coding. The video was assessed by a trained DB‐DOS coder who was unfamiliar with the child (and the child's clinical status). Coders (*N* = 5) were psychologists and behavioral scientists who were trained prior to performing the DB‐DOS. The training included studying the manual, assessing live and videotaped observations, and practicing coding in pairs. Coders coded 20 sessions during an 8‐month period. A level of agreement was reached in which scores would not deviate by more than one point on a maximum of 10 items. These two expert coders trained an independent team of coders (minimum level of education: bachelor's degree in psychology). To ensure reliable coding, interrater meetings were held every 2 weeks to discuss observational difficulties and potential differences in scores. Thereafter, coders worked independently and were blinded to the child's clinical diagnosis (i.e., ADHD presentation, presence of any comorbidities) and treatment status. Ten percent of the behavioral assessments were randomly selected and coded independently by two coders to monitor ongoing interrater reliability.

### Measurements

#### Disruptive behavior diagnostic observation schedule

The DB‐DOS was originally developed to assess disruptive behaviors in preschool children aged 3–5 years. The age‐adapted DB‐DOS used in the present study was developed by the present research team, building on the Utrecht preschool DB‐DOS framework used by Bunte et al. (2013), which was closely aligned with the original DB‐DOS developed by Wakschlag and colleagues. Prof. Walter Matthys, principal investigator of the Utrecht preschool DB‐DOS study, was also part of the present research team, ensuring continuity in DB‐DOS expertise. The present adaptation preserved the original DB‐DOS logic of pressing for clinically relevant disinhibited and disruptive behaviors, while adapting materials, rewards, instructions, and task demands to the developmental level of school‐aged children. Further details on the development and adaptation procedure are provided in Supporting Information S1: Appendix S1 and Table S1.

During the DB‐DOS assessment, structured parent‐ and examiner‐led tasks were used to elicit clinically relevant behaviors such as noncompliance, frustration, impulsivity, rule‐breaking, and anger. The adapted administration initially included three interactional contexts: the parent context (PC), the examiner context (EC), and the Parent‐and‐Examiner Context. Because the Parent‐and‐Examiner Context showed insufficient variability in atypical behavior, only the PC and EC were retained for scale construction and subsequent analyses. A detailed overview of the administration procedure, tasks, behavioral presses, and typically observed behaviors is provided in Supporting Information S1: Appendix S2 and Table S2.

Behaviors were coded separately for each context on a 4‐point scale ranging from 0 = normative behavior to 3 = markedly atypical behavior. The initial item pool included items derived from ADHD‐, ODD‐, and emotion dysregulation‐related behavioral domains. These labels refer to the origin of the item pool and not to diagnostic scales. After item selection and principal component analyses, the retained items were organized into two observational dimensions: BR and AM. The item‐selection procedure and final retained item set are described in Supporting Information S1: Appendix S3 and Tables S3 and S4.

#### Parent‐ and teacher ratings to examine external validity

In addition to the SWAN and SDQ (see Participants), parents and teachers were invited to complete the SDQ (Goodman, 1997). The SDQ is a short screening instrument (25 items) for emotional and behavioral problems in 4–17‐year‐old children. We used the Conduct Problems subscale to examine validity (parent items: Cronbach's *α* = 0.72, teachers: *α* = 0.68).

Parents and teachers further completed the Behavior Rating Inventory of Executive Function (BRIEF) (Gioia et al., 2000). The BRIEF consists of 75 items on behaviors related to executive functioning at home or school in 5–18‐year‐old children. We used the subscales “Emotion Regulation,” “Inhibition,” and “Working memory” to examine validity (Parents: *α* = 0.93, teachers: *α* = 0.96).

Parents and teachers filled out the CSBQ (Hartman et al., 2006). This is a 96‐item questionnaire that describes a broad range of severe and less severe behaviors typical of ASD. We used the total scale for the validity analyses (parents: *α* = 0.92, teachers: *α* = 0.93).

As negative parent‐child interactions are more common in children with ADHD and/or ODD, the Parenting Stress Questionnaire (PSQ) (Vermulst et al., 2011) and Abbreviated Scale for Parental Behavior (ASPB) (Van Leeuwen et al., 2013) were used. The PSQ consists of 34 items on stressors related to child upbringing experienced by parents. We used subscales “Parent‐Child relationship problems,” “Parenting problems” and “Parental role restriction” for validity analyses (Cronbach's *α* = 0.75). The ASPB consists of 25 items on parental behavior and style. We used positive parenting involvement and punishment (physical or otherwise) for the external validity analyses (Cronbach's *α* = 0.80).

### Statistical analyses

#### Item selection

Item selection was based on frequency and variance, interrater agreement, redundancy within contexts, cross‐context consistency, associations with parent‐ and teacher‐rated behavioral domains, principal component analysis (PCA) loadings, and conceptual fit. Items were rated and selected independently by three expert behavioral scientists (C.A.H., N.R., W.M.). Detailed selection criteria and decision rules are provided in Supporting Information S1: Appendix S3 and Table S3.

#### Psychometric qualities of the disruptive behavior diagnostic observation schedule scales

Principal component analysis was used to derive the scale scores for both PC and EC. To evaluate whether the two‐component solution was robust across age and gender, the PCA was repeated separately for boys and girls and for younger and older children based on a median split. Internal consistency reliability was examined using Cronbach's alpha (Cronbach, 1951). Interrater agreement at the domain level and context was assessed using intraclass correlation coefficients (ICC). Convergent validity was examined by computing Pearson's correlations for the DB‐DOS scale scores and parent and teacher ratings. We did not stratify or adjust the ICCs and convergent‐validity correlations by age or gender, because these analyses were intended to evaluate the overall psychometric performance of the DB‐DOS in the full clinical sample. Stratified analyses would have substantially reduced the available sample size, especially for girls and for analyses at follow‐up, and would have produced unstable estimates for correlations and ICCs. Age‐ and gender‐related patterns in DB‐DOS scores were therefore examined descriptively and interpreted cautiously.

To evaluate the psychometric qualities of the DB‐DOS scales administered at 5 weeks and 1 year compared to baseline, coded items were used for principal component analyses to evaluate scores within the PC and EC. Internal consistency reliability was examined using Cronbach's alpha (Cronbach, 1951). Inter‐rater agreement at the domain level and context was assessed using ICC. Convergent validity was examined by computing Pearson correlations for the DB‐DOS scale scores and the parent and teacher ratings at 5 weeks and 1 year.

DB‐DOS change scores were calculated by subtracting DB‐DOS scores at 5 weeks or 1 year from baseline scores. Similarly, parent and teacher change scores were computed by subtracting parent or teacher scores at 5 weeks or 1 year from parent or teacher scores at baseline. Pearson correlations were used to examine changes as observed in the DB‐DOS in relation to change, as reported by parents and teachers at 5 weeks and 1 year. Because children received treatment between assessments, stability coefficients and change scores across time cannot be interpreted as pure test‐retest reliability or as evidence of intervention effects alone. They may reflect measurement stability, true behavioral change, developmental change, repeated‐assessment effects, and other time‐related influences. IBM SPSS Statistics 25 was used for statistical analysis.

## RESULTS

### Item selection and retained contexts

After three iterations, 14 items were removed and two highly overlapping vigorous motor activity items were combined. The Parent‐and‐Examiner Context was excluded from scale construction because most items showed very little variation in this context. Most inattention items were removed because they were difficult to press and code reliably, whereas one emotion dysregulation item was retained because it contributed to the AM dimension without introducing redundancy. The final 19‐item set was used in the Parent and Examiner Contexts for subsequent analyses (see Supporting Information S1: Appendix S3 and Table S4).

### Dimensional structure

Principal component analyses showed that the retained item set was captured by two observational dimensions: behavioral regulation (BR; behaviors such as difficulties sustaining attention, excessive talking, and impulsive answers) and anger modulation (AM; behaviors such as defiance, aggression, and dysregulated negative affect). This two‐component structure held across the Parent and Examiner Contexts and was also observed at the 5‐week and 1‐year assessments. As a sensitivity check, the PCA was repeated after excluding the retained emotion dysregulation item; this yielded the same two‐component solution, supporting the robustness of the retained structure. Details are presented in Table 2.

**TABLE 2:** Principal component analyses of DB‐DOS items by interactional context and assessment wave.

Observed behaviors	Baseline				5 weeks				1 year			
	Behavioral regulation		Anger modulation		Behavioral regulation		Anger modulation		Behavioral regulation		Anger modulation	
	Parent context	Examiner context	Parent context	Examiner context	Parent context	Examiner context	Parent context	Examiner context	Parent context	Examiner context	Parent context	Examiner context
1. Defiance												
2. Passive noncompliance												
3. Predominance of noncompliance	0.31	0.29	0.54	0.48	0.03	0.16	0.72	0.69	0.04	−0.06	0.66	0.79
4. Rule breaking with supervision	0.43	0.41	0.28	0.13	0.46	0.37	0.28	0.10	−0.01	0.08	0.52	0.45
5. Rule breaking without supervision	0.39	0.18	0.11	0.12	0.49	0.16	0.15	−0.13	0.14	0.21	0.23	0.04
6. Lack of admission of rule breaking												
7. Provocative behavior	0.15	0.17	0.64	0.53	0.23	0.10	0.59	0.59	0.01	−0.05	0.67	0.80
8. Behavioral inflexibility	0.34	0.32	0.32	0.44	0.10	0.44	0.53	0.26	0.22	0.13	0.53	0.60
9. Destructiveness			0.59	0.52	0.06	0.03	0.58	0.64	0.03	0.20	0.59	0.41
10. Directed aggression	−0.01	0.12	0.57	−0.13	0.13	−0.23	0.42	0.47	**	**	**	**
11. Verbal aggression—Threats	−0.13	−0.36	0.65	0.82	**	**	**	**	**	**	**	**
12. Verbal aggression—Cursing			0.71	0.44	−0.11	−0.18	0.47	0.54	−0.15	0.19	0.49	0.03
13. Spiteful behavior	−0.22	−0.33	0.73	0.83	−0.23	−0.13	0.72	0.48	−0.30	−0.06	0.62	0.37
14. Sneaky behavior												
15. Intensity of emotional behavior												
16. Predominance of emotional behavior	−0.02	0.21	0.74	0.61	−0.03	0.12	0.74	0.66	0.08	0.04	0.73	0.66
17. Ease of elicitation of emotional behavior												
18. Rapid escalation of emotional behavior												
19. Difficulty recovering from emotional behavior												
20. Copes with emotions poorly												
21. Careless/inattention to details	0.22	0.41	0.12	−0.12	0.48	0.25	−0.01	−0.05	0.09	0.43	0.09	−0.21
22. Difficulty sustaining attention	0.17	0.40	−0.09	−0.01	−0.19	0.37	0.08	−0.01	0.46	−0.13	−0.17	0.38
23. Does not seem to listen												
24. Fails to follow through on tasks/instructions												
25. Easily bored	0.28	0.50	0.30	0.46	−0.22	−0.08	0.47	0.54	0.40	−0.10	0.01	0.62
26. Easily distracted												
27. Fidgets, squirms												
28. Difficulty remaining seated and vigorous motor activity	0.82*	0.60*	−0.14*	−0.04*	0.76	0.63*	−0.14	0.16*	0.25*	0.55*	0.50*	0.23*
29. Runs about/is on‐the‐go	0.82*	0.60*	−0.14*	−0.04*	0.76*	0.63*	−0.14*	0.16*	0.25*	0.55*	0.50*	0.23*
30. Difficulty playing quietly												
31. Talks excessively	0.37	0.62	0.11	0.07	0.20	0.78	0.26	−0.18	0.72	0.45	−0.10	0.26
32. Answers impulsively	0.36	0.68	−0.04	−0.14	0.19	0.81	−0.09	−0.19	0.69	0.89	−0.25	−0.14
33. Difficulty waiting (non verbal)	0.74	0.64	−0.09	0.22	0.78	0.67	−0.15	0.13	0.47	0.60	0.24	0.23
34. Interrupts, difficulty waiting (verbal)	0.75	0.74	−0.13	−0.04	0.40	0.79	0.24	−0.16	0.71	0.90	0.09	−0.20

*Note*: *item 28 and 29 were merged into 1 item. **Cells marked ** were omitted from the PCA because of very low variance.

### Reliability and descriptive scale characteristics

Table 3 presents the descriptive statistics and reliability estimates for the retained DB‐DOS scales at baseline. Interrater reliability was excellent across scales and contexts, and internal consistency ranged from acceptable to good. Mean scores were higher in the PC than in the EC, indicating that more disinhibited and disruptive behaviors were elicited in interaction with the parent.

**TABLE 3:** Descriptive statistics, internal consistency, and interrater reliability of DB‐DOS scales by interactional context at baseline.

Subscale	No. items	PC M (SD)	PC *α*	PC ICC	EC M (SD)	EC *α*	EC ICC	Total M (SD)
BR (0–27)	9	9.5 (3.8)	0.66	0.93	7.3 (4.5)	0.73	0.92	16.9 (7.1)
AM (0–30)	10	5.8 (4.8)	0.80	0.93	2.4 (3.2)	0.75	0.89	8.2 (7.0)
Total (0–57)	19	15.3 (7.5)	0.81	0.92	9.8 (6.7)	0.80	0.91	25.2 (12.4)

*Note:* Full sample *N* = 208, IRR subsample *N* = 20.

Abbreviations: AM = anger modulation, BR = behavioral regulation, EC = examiner context, ICC = intraclass correlation coefficient, PC = parent context, Total = sum of both contexts and scales, *α* = Cronbach’s alpha.

### Convergent validity

Associations between DB‐DOS scores and parent‐ and teacher‐rated behavioral domains are shown in Table 4. Correlations were generally small to moderate and were most consistent for DB‐DOS scores in the PC. This pattern provides partial support for convergent validity while also indicating that structured observation, parent reports, and teacher reports capture related but non‐identical perspectives on child behavior.

**TABLE 4:** Associations between DB‐DOS scale scores and parent‐ and teacher‐rated behaviors at baseline, 5 weeks, and 1 year.

	Measure		*N*	BR			AM			Total
				PC	EC	Total	PC	EC	Total	
B	SDQ	Conduct problems—parent	210	0.20	0.06	0.14	0.15	0.01	0.11	0.15
5W			204	**0.16**	0.11	**0.15**	**0.19**	0.09	**0.17**	**0.19**
1Y			162	0.12	0.11	0.12	**0.21**	0.01	0.10	0.13
B		Conduct problems—teacher	208	**0.24**	0.03	**0.15**	**0.20**	0.07	**0.18**	**0**.**19**
5W			199	**0.20**	**0.19**	**0.22**	**0.17**	**0.15**	**0.20**	**0.24**
1Y			162	0.08	0.11	0.11	0.13	0.01	0.12	0.12
B		Hyperactivity—parent	210	0.05	0.09	0.08	0.02	0.05	0.04	0.07
5W			204	**0.20**	**0.16**	**0.21**	0.12	**0.18**	**0.17**	**0.22**
1Y			162	0.10	**0.20**	**0.16**	0.12	**0.17**	**0.15**	**0.18**
B		Hyperactivity—teacher	208	**0.21**	**0.14**	**0.21**	0.08	0.09	0.09	**0.17**
5W			199	**0.21**	**0.21**	**0.24**	**0.15**	0.12	**0.16**	**0.23**
1Y			162	0.17	0.16	**0.19**	0.14	**0.19**	**0.19**	**0.21**
B	BRIEF	Inhibition—parent	181	**0**.**23**	**0.24**	**0.28**	**0.23**	**0.15**	**0.22**	**0.29**
5W			171	0.02	0.02	0.02	0.07	0.04	0.06	0.05
1Y			162	0.11	0.01	0.05	0.13	**0.18**	0.15	0.10
B		Inhibition—teacher	178	**0.27**	**0.18**	**0.27**	**0**.**23**	0.13	**0.22**	**0.28**
5W			165	0.09	**0.20**	**0.17**	0.02	**0.15**	0.09	**0.15**
1Y			162	**0.23**	0.07	0.17	**0.25**	−0.02	0.14	0.17
B		Emotional control—parent	181	0.06	−0.07	−0.01	0.12	−0.02	0.07	0.04
5W			171	0.06	0.04	0.06	0.10	0.14	0.14	0.11
1Y			162	**0.18**	0.14	**0.19**	**0.20**	**0.24**	**0.26**	**0.24**
B		Emotional control—teacher	178	**0.15**	0.08	0.13	**0.22**	**0.15**	**0.22**	**0.20**
5W			165	0.11	0.12	0.13	0.08	0.14	0.12	0.14
1Y			151	0.08	0.09	0.10	0.12	−0.06	0.03	0.08
B		Working memory—parent	181	0.02	**0.14**	0.10	−0.02	0.02	−0.01	0.05
5W			180	0.03	0.11	0.08	−0.03	0.09	0.02	0.06
1Y			162	0.14	0.05	0.11	0.13	0.15	0.16	0.15
B		Working memory—teacher	178	0.06	0.14	0.12	0.07	0.06	0.08	0.11
5W			171	0.07	0.13	0.12	−0.04	0.05	0.001	0.07
1Y			162	**0.24**	0.06	0.18	0.16	−0.03	0.10	0.16
B	PSQ	Parent‐child relationship problems	212	0.08	−0.02	0.03	0.09	0.05	0.09	0.07
5W			208	−0.04	0.03	−0.003	−0.02	0.002	−0.02	−0.01
1Y			162	−0.11	−0.04	−0.08	**−0.26**	0.04	−0.13	−0.11
B		Parenting problems	212	0.07	0.005	0.04	0.08	0.06	0.08	0.07
5W			208	−0.04	0.005	−0.02	−0.10	−0.02	−0.08	−0.05
1Y			162	−0.04	0.01	−0.01	**−0.17**	0.07	−0.06	−0.03
B		Parental role restriction	212	**0.18**	**0.19**	**0.22**	**0.20**	**0.16**	**0.21**	**0.25**
5W			208	0.12	0.10	0.12	0.05	0.08	0.07	0.11
1Y			162	**0.27**	**0.22**	**0.28**	**0.27**	**0.26**	**0.30**	**0.32**
B	BSPB	Positive parenting	180	0.07	**0.17**	**0.15**	<0.01	−0.02	−0.009	0.08
5W			177	0.05	0.10	0.09	−0.02	0.11	0.03	0.07
1Y			148	0.04	0.08	0.08	0.006	0.16	0.07	0.09
B		Negative control	180	0.08	−0.01	0.03	0.08	−0.02	0.04	0.04
5W			177	−0.04	0.04	0.007	0.06	−0.02	0.02	0.01
1Y			148	0.03	0.02	0.02	0.02	0.07	0.08	0.05
B	CSBQ	Total score—parent	212	**0.18**	**0.21**	**0.23**	**0.21**	**0.25**	**0.26**	**0.28**
5W			204	0.09	**0.17**	**0.16**	**0.15**	**0.24**	**0.22**	**0.22**
1Y			162	**0.20**	**0.24**	**0.25**	**0.30**	**0.33**	**0.35**	**0.33**
B		Total score—teacher	178	**0.23**	**0**.**24**	**0.27**	0.13	**0.15**	**0.16**	**0.25**
5W			169	**0.17**	**0.31**	**0.28**	0.05	**0.16**	0.11	**0.23**
1Y			151	**0.29**	**0.29**	**0.34**	**0.33**	**0.22**	**0.35**	**0.38**

*Note*: Bold values indicate *p* < .05. Orange: Behavioral Regulation items, Blue: Anger Modulation items, Yellow: total of BR and AM scores. Darker colors indicate stronger correlations (c.q. heatmap).

Abbreviations: AM = Anger Modulation scale, BR = Behavioral Regulation scale, BRIEF = Behavior Rating Inventory of Executive Function, BSPB = Brief Scale of Parental Behavior, CSBQ = Children's Social Behavior Questionnaire, EC = examiner context, PC = parent context, PSQ = Parenting Stress Questionnaire, SDQ = Strengths and Difficulties Questionnaire.

### Repeated administration and scale stability

**FIGURE 1:**
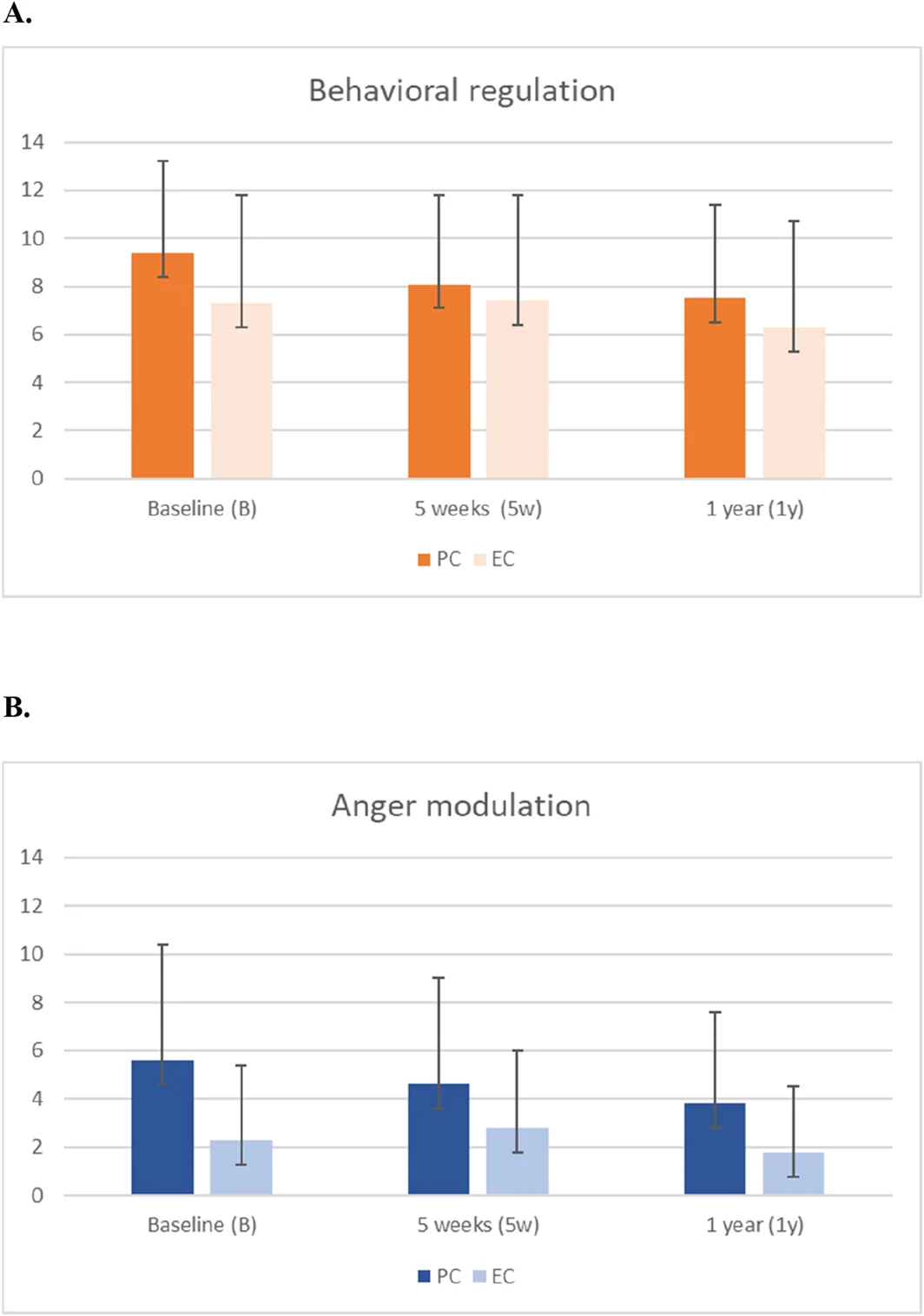
Mean DB‐DOS behavioral regulation and AM scores across assessment waves by context. Panel A presents BR scores; Panel B presents AM scores. Scores are shown separately for the PC and EC across assessment waves. Error bars indicate standard deviations. AM, anger modulation; BR, behavioral regulation; DB‐DOS, disruptive behavior diagnostic observation schedule; EC, examiner context; PC, parent context.

The repeated assessments showed a broadly comparable psychometric pattern across baseline, 5 weeks, and 1 year. Mean BR and AM scores by context and assessment wave are shown in Table 5 and Figure 1. Stability coefficients across repeated assessments are shown in Table 6. Because children received treatment between assessments, these coefficients should not be interpreted as pure test‐retest reliability; rather, they reflect both measurement stability and potential change over time. Exploratory checks showed a comparable PCA pattern for boys and girls and for younger and older children based on a median split. Descriptively, boys scored higher than girls on both BR and AM, and BR and AM were negatively associated with age, indicating fewer observable BR and AM behaviors among older than younger children.

**TABLE 5:** Mean DB‐DOS behavioral regulation and anger modulation scores by context and assessment wave.

DB‐DOS scale/wave	Parent context M (SD)	Examiner context M (SD)	Comparison
Behavioral regulation			
Baseline (B)	9.4 (3.8)	7.3 (4.5)	PC > EC
5 weeks (5W)	8.1 (3.7)	7.4 (4.4)	PC > EC
1 year (1Y)	7.5 (3.9)	6.3 (4.4)	PC > EC
Within‐context comparison	*B* = 5W > 1Y	*B* = 5W > 1Y	
Anger modulation			
Baseline (B)	5.6 (4.8)	2.3 (3.1)	PC > EC
5 weeks (5W)	4.6 (4.4)	2.8 (3.2)	PC > EC
1 year (1Y)	3.8 (3.8)	1.8 (2.7)	PC > EC
Within‐context comparison	*B* > 5W = 1Y	5W > *B* > 1Y	

*Note*: Values indicate mean DB‐DOS scores (SD).

Abbreviations: EC = examiner context, PC = parent context.

**TABLE 6:** Stability coefficients for DB‐DOS scale scores across repeated assessments.

Context/wave	BR ICC	BR 95% CI	AM ICC	AM 95% CI	Total ICC	Total 95% CI
Parent context: B‐5W	0.61	0.49–0.71	0.63	0.51–0.72	0.70	0.60–0.77
Parent context: B‐1Y	0.57	0.41–0.69	0.51	0.32–0.64	0.64	0.51–0.74
Examiner context: B‐5W	0.77	0.69–0.82	0.69	0.59–0.76	0.80	0.74–0.85
Examiner context: B‐1Y	0.77	0.68–0.83	0.62	0.47–0.72	0.77	0.68–0.83
Total: B‐5W	0.76	0.68–0.82	0.70	0.60–0.77	0.79	0.72–0.84
Total: B‐1Y	0.77	0.68–0.83	0.62	0.48–0.72	0.78	0.69–0.84

*Note*: 95% confidence intervals.

Abbreviations: AM = anger modulation, BR = behavioral regulation, EC = examiner context, ICC = intraclass correlation coefficient, PC = parent context.

### Change over time

To examine whether the DB‐DOS captured change over time, change scores on the DB‐DOS were correlated with change scores based on parent and teacher reports (SWAN and SDQ). Parent‐reported change at 5 weeks correlated modestly with parent‐reported change at 1 year, whereas teacher‐reported change at 5 weeks did not correlate with teacher‐reported change at 1 year. Change observed with the DB‐DOS at 5 weeks correlated moderately with change observed with the DB‐DOS at 1 year. Parent‐ and teacher‐reported change were generally not correlated, except for change in disruptive behavior at 1 year. Changes in AM at 1 year observed in the DB‐DOS PC correlated significantly with teacher‐rated changes in AM. Several other small correlations between DB‐DOS and questionnaire change scores were in the expected direction and domain but did not reach significance (Table 7).

**TABLE 7:** Associations between DB‐DOS change scores and parent‐ and teacher‐rated change scores across repeated assessments.

		ΔB‐1Y		ΔB‐54W	
Domain		pHI	tHI	PC	EC
Hyperactivity ‐ impulsivity	SWAN: Parent HI (pHI)	0.50*	−0.03	0.001	−0.07
	SWAN: Teacher HI (tHI)	0.05	0.06	0.001	0.13
	DB‐DOS—BR: Parent context (PC)	0.001	0.12	0.48*	0.34*
	DB‐DOS—BR: Examiner context (EC)	0.01	−0.04	0.25*	0.55*
		**pED**	**tED**	**PC**	**EC**
Anger modulation	SDQ: Parent ED (pED)	0.49*	0.04	0.06	0.01
	SDQ: Teacher ED (tED)	0.21*	0.14*	0.04	0.04
	DB‐DOS—AM: Parent context (PC)	0.20*	0.09	0.62*	0.39*
	DB‐DOS—AM: Examiner context (EC)	0.11	−0.06	0.42*	0.46*

*Note*: Scores above the grey diagonal represent change scores from baseline to 5 weeks (Δ*B*‐5W); scores below represent change scores from baseline to 1 year (Δ*B*‐1Y).

Abbreviations: AM = anger modulation, BR = behavioral regulation, EC = examiner contextm, PC = parent context.

*Indicates significant correlations *p* < 0.05.

## DISCUSSION

We adapted the DB‐DOS for use with children aged 5–12 years and examined its reliability, validity, and ability to capture change over time. The retained item set yielded two observational dimensions, BR and AM, which could be reliably coded in both the PC and EC. The Parent‐and‐Examiner Context elicited too little variation in atypical behavior and was therefore not retained for scale construction or subsequent analyses. Across retained contexts, interrater reliability was excellent, internal consistency was acceptable to good, and associations with parent and teacher reports provided partial support for convergent validity. These associations were generally modest, which is consistent with the expectation that structured observations, parent reports, and teacher reports capture related but non‐identical perspectives on child behavior.

### Two components

The two‐component solution was comparable to the dimensions reported for the preschool DB‐DOS, supporting continuity between the preschool instrument and the age‐adapted version (Bunte et al., 2013; Wakschlag et al., 2005, Wakschlag, Briggs‐Gowan, et al., 2008, Wakschlag, Hill et al., 2008). Additional exploratory checks showed a comparable PCA pattern for boys and girls and for younger and older children, supporting the robustness of the scale structure. Descriptively, boys showed higher BR and AM scores than girls, and older children showed fewer observable BR and AM behaviors than younger children. These patterns are consistent with the clinical expectation that overt disinhibited and disruptive behaviors may be more readily observable in boys and younger children. Because the present study was designed to evaluate the DB‐DOS in the full clinical sample, and because further stratification would have produced small and unstable estimates, these subgroup patterns should be interpreted cautiously.

The initial item pool was informed by ADHD‐, ODD‐, and emotion dysregulation‐related behaviors, but the final scales should be interpreted as observational dimensions of BR and AM rather than as disorder‐specific ADHD or ODD scales. This distinction is important because disinhibited and disruptive behaviors cut across diagnostic categories. The associations between DB‐DOS scores and informant‐rated ADHD, ODD/conduct, and ASD‐related behaviors are consistent with the interrelatedness of BR, AM, and broader neurodevelopmental functioning (Evans et al., 2024; Matthys & Lochman, 2017; Rommelse et al., 2015). Relatively strong associations with ASD characteristics may reflect the fact that the DB‐DOS is a structured social interaction assessment containing unexpected, frustrating, and disliked elements that require children to adapt their behavior and expectations. Elevated DB‐DOS scores should therefore be interpreted within a broader clinical assessment and not as indicative of a specific diagnosis. Future studies could examine whether the DB‐DOS adds information to ASD assessments such as the ADOS, especially because the ADOS includes only an EC (Hampton et al., 2021).

### Parent and examiner context

The PC consistently elicited more disinhibited and disruptive behaviors than the EC. This pattern is consistent with previous DB‐DOS research showing contextual variation in observed disruptive behavior (Bunte et al., 2013; Petitclerc et al., 2015; Wakschlag et al., 2005, Wakschlag, Briggs‐Gowan, et al., 2008, Wakschlag, Hill et al., 2008). The higher mean levels in the PC likely reflect the ecological salience of parent‐child interaction and the familiarity of the parent‐child relationship. However, the EC remains clinically meaningful. Although problem behaviors are generally less frequent in interaction with an unfamiliar examiner, their occurrence may indicate more pervasive behavioral dysregulation and may therefore provide complementary information (Belden et al., 2008; Petitclerc et al., 2015; Wakschlag et al., 2012; van den Akker et al., 2022). The combined use of Parent and Examiner Contexts therefore appears important for capturing both ecologically salient behavior and behavior that generalizes beyond the parent‐child relationship.

### Inattentive presentation of ADHD

The results also clarify the limits of clinic‐based observation for predominantly inattentive presentation of ADHD. Most inattention items did not survive item selection because they were difficult to press for and code reliably from the available video recordings. Behaviors such as “does not seem to listen” and “difficulty sustaining attention” may require information that is not readily observable from a single camera angle and without close‐up facial information. This finding aligns with the broader challenge of observing attention regulation directly, whether in clinic‐based or school‐based assessments (Gadow et al., 1996; Shapiro & Heick, 2004). With respect to the DB‐DOS, the clinical value appears strongest for overt disinhibited and disruptive behaviors rather than for isolated attention regulation problems without concurrent behavioral dysregulation.

### Change over time

The repeated assessments provide preliminary evidence that the DB‐DOS can capture change over time while retaining comparable psychometric properties. Change measured with the DB‐DOS showed more correspondence with parent‐ or teacher‐reported change than parent‐ and teacher‐reported change showed with each other. This supports the value of structured observation as an additional perspective on behavioral change, particularly in intervention contexts where treatment allocation cannot be blinded (Brotman et al., 2008; Gardner et al., 2006; Menting et al., 2013; Narad et al., 2015; Posthumus et al., 2012). However, these findings should not be interpreted as pure test‐retest reliability or as direct evidence of treatment effects. Because children received treatment between assessments and no untreated repeated‐assessment comparison group was available for the present psychometric analyses, change scores may reflect a combination of treatment‐related change, natural development, regression to the mean, repeated‐assessment effects, and contextual variation.

### Strengths and weaknesses

Strengths of this study include the large clinical sample of children with ADHD, the standardized and blinded coding of videotaped observations, the availability of parent and teacher reports, and repeated assessments across both short‐ and longer‐term follow‐up. The sample was clinically well characterized through prior clinical diagnosis, research‐based diagnostic information from parent interviews and teacher ratings, and assessment of comorbid behavioral, neurodevelopmental, and internalizing problems. A general limitation is that participation required willingness to take part in a clinical trial. However, willingness to follow dietary treatment was not a prerequisite for participation, as children with ADHD were eligible regardless of treatment condition. In addition, although exploratory findings suggested comparable scale structure across age and gender subgroups, future studies should examine developmental and gender‐related differences in DB‐DOS scores in larger and more balanced samples. In future research we will involve a typically developing group and determine cutoff points of ADHD symptom scores and ODD/CD symptom scores, which divide clinical cases from typically developing cases.

## CONCLUSION

Taken together, this study shows that disinhibited and disruptive behaviors can be elicited and reliably coded in 5–12‐year‐old children with ADHD using the age‐adapted DB‐DOS. The retained item set yielded two robust observational dimensions, BR and AM. The DB‐DOS should not be interpreted as a stand‐alone diagnostic instrument, but may provide clinically valuable observational information to complement parent and teacher reports in diagnostic assessment and intervention research. Further research is needed to examine normative reference data, discriminative validity in typically developing and clinical comparison groups, utility in ODD/CD‐only samples, and applicability in older children and adolescents.

## AUTHOR CONTRIBUTIONS

**Margreet Bierens**: Data curation; formal analysis; investigation; project administration; validation; visualization; writing—original draft; writing—review and editing. **Annick Huberts‐Bosch**: Data curation; investigation; project administration; writing—review and editing. **Verena Ly**: Investigation; project administration; writing—review and editing. **Walter Matthys**: Conceptualization; methodology; supervision; writing—review and editing. **Jan Buitelaar**: Conceptualization; funding acquisition; methodology; supervision; writing—review and editing. **Catharina A. Hartman**: Conceptualization; methodology; supervision; writing—review and editing. **Nanda Rommelse**: Conceptualization; funding acquisition; investigation; methodology; project administration; supervision; validation; writing—review and editing.

## CONFLICT OF INTEREST STATEMENT

The authors declare no conflicts of interest.

## ETHICAL CONSIDERATIONS

The study was approved by the local medical ethics Committee on Research involving Human Subjects (CMO), the Institutional Review Board of Karakter Child and Adolescent Psychiatry and Radboudumc, approval number: 2014‐1349, date: January 22, 2015. Written informed consents were obtained. This study was conducted in accordance with the principles of the Declaration of Helsinki.

## Supporting information

Supporting Information S1

## Data Availability

The data supporting the findings of this study are available from the corresponding author upon reasonable request.
