# Supplementary material for: Reliability, validity, and change over time in disinhibited and disruptive behaviors assessed with the age‐adapted disruptive behavior diagnostic observation schedule in children aged 5–12 years with ADHD
Source: JCPP Adv. 2026 Sep 16:e70149. Online ahead of print. doi: 10.1002/jcv2.70149 (PMC13579545; doi:10.1002/jcv2.70149)
Supplement: Supplementary file 1 [file JCV2-9999-e70149-s001.docx]

**Reliability, validity, and change over time in disinhibited and disruptive behaviors assessed with the age-adapted Disruptive Behavior Diagnostic Observation Schedule (DB-DOS) in children aged 5–12 years with ADHD**

# Supporting Information

# Appendix S1. Development and adaptation of the DB-DOS^5–12^

The DB-DOS^5–12^ was developed by the present research team, building on the Utrecht preschool DB-DOS framework used by Bunte et al. (2013), which had been closely aligned with the original DB-DOS developed by Wakschlag and colleagues. Prof. Walter Matthys, principal investigator of the Utrecht team that closely collaborated with Wakschlag's group during the development and implementation of the preschool version, was also part of the present research team. This ensured continuity in DB-DOS expertise. The present 5-12-year adaptation did not involve new direct training or close collaboration with Wakschlag's team. Rather, it built on the established DB-DOS framework, the Utrecht preschool adaptation, and the expertise of team members with prior involvement in that work.

The main aim of the age adaptation was to preserve the original DB-DOS assessment logic while making the tasks developmentally appropriate for school-aged children. Materials, rewards, instructions, and task demands were adapted for younger school-aged children aged 5-8 years and older school-aged children aged 9-12 years. The adapted version retained the central principle of pressing for clinically relevant disinhibited and disruptive behaviors across structured interactional contexts. The Parent-and-Examiner Context was initially included in the DB-DOS^5–12^ administration but was not retained for scale construction because it showed insufficient variability in atypical behavior.

**Table S1.** Overview of adaptations from the preschool DB-DOS to the DB-DOS^5–12^

| **Adaptation domain** | **Preschool DB-DOS** | **DB-DOS^5–12^** | **Rationale** |
| --- | --- | --- | --- |
| Target age range | Preschool children aged 3-5 years | School-aged children aged 5-12 years | To extend standardized clinic-based observation to an older age group. |
| Rewards/prizes | Age-appropriate toys and candy | Age-appropriate toys and small monetary/prize incentives | To maintain motivational salience for school-aged children. |
| Instructions | Primarily directed toward the parent in several tasks | More directly addressed to the child | To match the greater autonomy and rule understanding of school-aged children. |
| Task duration | Short preschool tasks, typically around 2 minutes | Tasks typically lasted 4 minutes; the PEC interview task lasted 8 minutes | To provide sufficient opportunity to observe behavior in older children. |
| Task difficulty | Preschool materials and activities | More challenging materials, age-specific variants for ages 5-8 and 9-12 years, and parallel versions across waves | To preserve behavioral presses while preventing tasks from becoming too easy or too childish. |
| Parent Context | Parent-child tasks designed to elicit noncompliance, frustration, and rule-related behavior | Parent-child tasks adapted with age-appropriate play, sorting, waiting, and impossible tasks | To maintain ecological salience of parent-child interaction. |
| Examiner Context | Examiner-led tasks with preschool-appropriate materials | Examiner-led tasks adapted to elicit waiting, frustration, impulsivity, rule-breaking, and behavioral inflexibility | To observe behavior with an unfamiliar adult under standardized conditions. |
| Parent-and-Examiner Context | Not part of the original preschool structure in the same form | Initially included as a context in which the child waited or played while parent and examiner interacted | Explored as an additional context, but not retained because of insufficient variability in atypical behavior. |

# Appendix S2. DB-DOS^5–12^ administration and task descriptions

The DB-DOS^5–12^ was administered in two consecutive modules that were retained for scoring: the Parent Context and the Examiner Context. Each task lasted approximately 4 minutes. At the start of the Parent Context, the parent was invited to the examination room individually to receive standardized instructions. The examiner then welcomed the child and parent and explained the single rule in the room to the child: "You are not allowed to touch or play with the prizes and toys. Good luck!" The examiner then left the room and observed from behind the one-way screen.

In the Parent Context, the child completed five tasks in interaction with the parent. Support was gradually reduced across tasks. After the parent-phone task, the parent repeated the rule, left the room, and joined the examiner behind the one-way screen while the child waited or played alone. This task was included to probe rule-breaking and impulsive behavior in the presence of attractive but off-limits toys and prizes.

The Examiner Context started when the examiner re-entered the room after the child-alone task and asked whether the child had touched or played with the prizes or toys. The child then completed five tasks in interaction with the examiner. Standardized scripts and responses were used to maintain comparable behavioral presses across children. For example, during the defective-toy task the examiner prompted the child to keep trying and used a standardized warning if aggressive behavior occurred. Parallel versions of tasks and materials were used across baseline, 5-week, and 1-year assessments to reduce repetition effects while preserving the same behavioral presses.

**Table S2.** DB-DOS^5–12^ tasks, administration details, behavioral presses, and typically observed behaviors

| **Context** | **Task** | **Administration details / age adaptation** | **Behavioral press** | **Typically observed behaviors** |
| --- | --- | --- | --- | --- |
| Parent Context | Building blocks / play | Parent and child are seated at the table. A large box with colored building blocks is presented, and parent and child are invited to play and build together. | Observe social play and parent-child interaction | Reciprocity, dominance, bossy behavior, individual versus interactive play |
| Parent Context | Sort blocks by color | The parent tells the child to stop playing and sort the blocks by color into four trays (5-8 years) or six trays (9-12 years). | Elicit noncompliance and task-persistence difficulties | Noncompliance, passive noncompliance, careless responding, difficulty remaining seated |
| Parent Context | Impossible maze / puzzle | The child is given an impossible maze or puzzle. The parent tells the child that completing it in time will earn a prize. After 4 minutes, the parent tells the child that time is up and no prize was won; the parent may comfort the child as at home. | Elicit frustration and dysregulated behavior | Fidgeting, help-seeking, crying, anger, verbal aggression |
| Parent Context | Parent on the phone | The child receives paper and a pencil (5-8 years) or no materials (9-12 years). The parent makes a 4-minute phone call with the examiner and instructs the child not to disturb. | Elicit noncompliance, provocation, and impulsive behavior | Interrupting, provocative behavior, rule-breaking, verbal aggression |
| Parent Context | Child waits / plays alone | After repeating the room rule, the parent leaves to join the examiner behind the one-way screen. The child waits or plays alone for 4 minutes with off-limits toys and prizes present. | Elicit rule-breaking and impulsive behavior | Rule-breaking, vigorous motor activity, fidgeting, talking or singing alone |
| Examiner Context | Draw circles / alphabet | The examiner asks the child to draw as many circles as possible (5-8 years) or all upper- and lower-case letters of the alphabet (9-12 years), stopping only when instructed. | Elicit noncompliance and task-persistence difficulties | Passive noncompliance, careless responding, difficulty sustaining attention or remaining seated |
| Examiner Context | Defective toy | The child receives a defective toy while the examiner plays with a functioning version for 2 minutes. The examiner uses scripted prompts, exchanges toys in the third minute, and returns the defective toy in the fourth minute. | Elicit frustration, provocation, and impulsive turn-taking | Anger, crying, verbal aggression, impulsive turn-taking |
| Examiner Context | Impossible game task | The child attempts an impossible ring-throwing or bow-and-arrow task to win a prize. The examiner leaves the room and returns after 4 minutes to tell the child that the prize was not won. | Elicit frustration, rule-breaking, and emotion dysregulation | Vigorous motor activity, anger, crying, verbal aggression, rule-breaking |
| Examiner Context | Drawing assignment | The child is asked to draw four objects while the examiner walks around, hums, and moves blocks back into the box as mild distractions. | Elicit noncompliance and task-persistence difficulties | Passive noncompliance, carelessness, distractibility, difficulty remaining seated |
| Examiner Context | Parlor game | The examiner explains a board/parlor game and takes deliberately long turns. | Observe interaction, waiting, impulsivity, and behavioral inflexibility | Impulsive turn-taking, cursing, rule-breaking, behavioral inflexibility |

Note. All tasks lasted approximately 4 minutes. Parallel task versions and materials were used across repeated assessments to preserve the same behavioral presses while reducing repetition effects.

## Appendix S3. Item-selection procedure

The initial item pool included items derived from ADHD-, ODD/CD-, and emotion dysregulation-related behavioral domains. These labels refer to the origin of the item pool and not to diagnostic scales. Items were rated and selected independently by three expert behavioral scientists (CH, NR, WM). Selection was based on frequency and variance, interrater agreement, redundancy, associations with parent- and teacher-rated behavioral domains, PCA loadings, and conceptual fit with the emerging dimensions.

**Table S3.** Item-selection criteria and decision rules

| **Criterion** | **Decision rule / rationale** |
| --- | --- |
| Frequency and variance | The item was scored as present in at least 5% of the sample and showed sufficient variance. |
| Interrater agreement | The item showed adequate coder agreement. |
| Redundancy | Items with very high correlations within the same context were flagged; when content overlapped, items were removed or combined. |
| Problematic negative correlations | Items with negative correlations inconsistent with their content or with similar parent-/teacher-rated domains were flagged. |
| External association | The item correlated at least .15 with one or more parent- or teacher-rated scales assessing related behavioral domains. |
| PCA loading and conceptual fit | Items were evaluated for fit with the emerging PCA-derived dimensions in the Parent Context and Examiner Context. |
| Expert review | Final decisions considered empirical results and conceptual fit with Behavioral Regulation and Anger Modulation. |

Following three iterations, 14 items were removed and two highly overlapping items on vigorous motor activity were combined. The Parent-and-Examiner Context was excluded from scale construction because most items showed very little variation. Most inattentive behavior items were removed because they were difficult to press and code reliably. Highly overlapping emotion dysregulation items were reduced to one retained item. An additional robustness check showed that the two-component structure remained unchanged when this retained emotion dysregulation item was excluded. The final 19-item set was used for PCA in the Parent Context and Examiner Context. As a sensitivity check, the PCA was repeated after excluding the retained emotion dysregulation item. This yielded the same two-component solution, supporting the robustness of the retained structure.

**Table S4.** Final DB-DOS^5–12^ item set after item selection

| **Behavioral Regulation** | **Anger Modulation** |
| --- | --- |
| 4. Rule-breaking with supervision | 3. Predominance of noncompliance |
| 5. Rule-breaking without supervision | 7. Provocative behavior |
| 21. Careless / inattention to details | 8. Behavioral inflexibility |
| 22. Difficulty sustaining attention | 9. Destructiveness |
| 28-29. Difficulty remaining seated / vigorous motor activity / runs about or is on the go | 10. Directed aggression |
| 31. Talks excessively | 11. Verbal aggression - threats |
| 32. Answers impulsively | 12. Verbal aggression - cursing |
| 33. Difficulty waiting (nonverbal) | 13. Spiteful behavior |
| 34. Interrupts / difficulty waiting (verbal) | 16. Predominance of emotional behavior |
|  | 25. Easily bored |

Note. The final item set included 19 items coded in two interactional contexts: Parent Context and Examiner Context.
